# Impact of germline DNA repair gene variants on prognosis and treatment of men with advanced prostate cancer

**DOI:** 10.1038/s41598-023-46323-5

**Published:** 2023-11-06

**Authors:** Emma B. Hansen, Questa Karlsson, Susan Merson, Sarah Wakerell, Reshma Rageevakumar, Jørgen B. Jensen, Michael Borre, Zsofia Kote-Jarai, Rosalind A. Eeles, Karina D. Sørensen

**Affiliations:** 1 Department of Molecular Medicine (MOMA), Aarhus University Hospital, Aarhus, Denmark; 2 Department of Clinical Medicine, Aarhus University, Aarhus, Denmark; 3 Division of Genetics & Epidemiology, The Institute of Cancer Research, London, UK; 4 Department of Urology, Regional Hospital of West Jutland, Gødstrup Hospital, Gødstrup, Denmark; 5 Department of Urology, Aarhus University Hospital, Aarhus, Denmark; 6 Royal Marsden NHS Foundation Trust, London, UK

**Keywords:** Prognostic markers, Prostate cancer, Genetics research, Outcomes research, Molecular medicine

## Abstract

The clinical importance of germline variants in DNA repair genes (DRGs) is becoming increasingly recognized, but their impact on advanced prostate cancer prognosis remains unclear. A cohort of 221 newly diagnosed metastatic castration-resistant prostate cancer (mCRPC) patients were screened for pathogenic germline variants in 114 DRGs. The primary endpoint was progression-free survival (PFS) on first-line androgen signaling inhibitor (ARSI) treatment for mCRPC. Secondary endpoints were time to mCRPC progression on initial androgen deprivation therapy (ADT) and overall survival (OS). Twenty-seven patients (12.2%) carried a germline DRG variant. DRG carrier status was independently associated with shorter PFS on first-line ARSI [HR 1.72 (1.06–2.81), *P* = 0.029]. At initiation of ADT, DRG carrier status was independently associated with shorter progression time to mCRPC [HR 1.56, (1.02–2.39), *P* = 0.04] and shorter OS [HR 1.99, (1.12–3.52), *P* = 0.02]. Investigating the contributions of individual germline DRG variants on PFS and OS revealed *CHEK2* variants to have little effect. Furthermore, prior taxane treatment was associated with worse PFS on first-line ARSI for DRG carriers excluding *CHEK2* (*P* = 0.0001), but not for noncarriers. In conclusion, germline DRG carrier status holds independent prognostic value for predicting advanced prostate cancer patient outcomes and may potentially inform on optimal treatment sequencing already at the hormone-sensitive stage.

## Introduction

The clinical importance of germline mutations in DNA repair genes (DRGs) in prostate cancer (PC) has become increasingly recognized in recent years. Multiple germline DRG variants have been associated with an increased risk of PC, including *BRCA1/2*, *ATM*, *CHEK2*, and specific mismatch repair gene variants^1–7^. In addition, the prevalence of germline DRG variants is significantly higher in metastatic PC (12–16%) compared to localized PC (5%) and to the general population (3%)^5,8,9^. Consistently, germline DRG variants have been associated with more aggressive phenotypes and poor outcome in localized and metastatic PC^10–13^, with prior studies primarily focusing on *BRCA1/2* and *ATM*. The National Comprehensive Cancer Network and the European Association of Urology now recommend germline testing of selected DNA repair genes in men with metastatic PC^14,15^. However, it remains unclear how the presence of specific germline DRG variants should impact on disease management.

Recent studies have centered on the impact of germline DRG variants on the outcomes of patients with metastatic castration-resistant PC (mCRPC)^8,9,16^. However, contradicting results have been published, particularly, on the impact of germline DRG variants on the outcomes of mCRPC patients receiving second-generation androgen signaling inhibitors (ARSI)^8,9,16^. Thus, there is a need for more knowledge about the influence of germline DRG variants on the prognosis and treatment of mCRPC patients.

In this study, we therefore aimed to investigate the prognostic significance of germline DRG variants for patients with mCRPC, including outcomes from initiation of primary androgen deprivation therapy (ADT) prior to castration resistance.

## Results

### Patients and germline DNA repair gene variants

**Figure 1:**
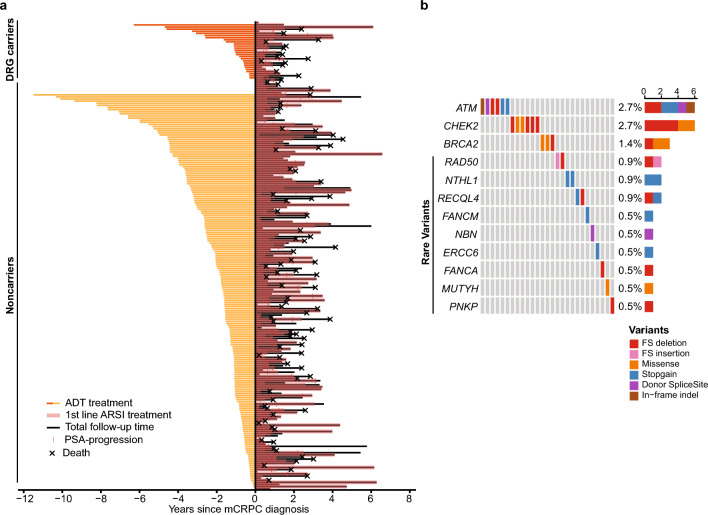
Patients and germline DNA repair gene variants. (**a**) Swimmer plot showing an overview of disease progression, ordered by DRG carrier status and time from initiation of ADT to mCRPC diagnosis. (**b**) Distribution of the pathogenic/likely pathogenic germline variants identified in the cohort. *DRG* DNA-repair genes, *ADT* Androgen deprivation therapy, *ARSI* androgen signaling inhibitor, *PSA* prostate-specific antigen, *FS* Frameshift.

We screened a consecutive cohort of 221 men with newly diagnosed mCRPC for pathogenic germline variants in coding regions of 114 DNA repair genes (Supplementary Table S1). Patients were included at two hospitals in Denmark (2016–2020) when starting first-line mCRPC treatment. At mCRPC diagnosis, median age was 73.2 years (IQR 68.5–78.5) and 81.9% (n = 181/221) had bone metastases (Table 1). As first-line mCRPC treatment, 98.6% (n = 218/221) of patients received ARSI (n = 195/221, 88.2% enzalutamide; n = 23/221, 10.4% abiraterone) treatment, while three patients received a taxane (n = 3/221, 1.4% docetaxel) (Table 1). At the last follow-up, 55.5% (n = 121/218) of patients had experienced prostate-specific antigen (PSA) progression on first-line ARSI and 43.9% (n = 97/221) of all patients had died (Table 1, Fig. 1A).

**Table 1:** Patient baseline characteristics and summary of prior and current treatments for localized, advanced, and castration-resistant prostate cancer.

Clinical variable	Full cohort	Carriers	Noncarriers	*P* value
	(n = 221)	(n = 27, 12.2%)	(n = 194, 87.8%)	
Median age at baseline, years (IQR)	73.2 (68.5–78.5)	76.1 (70.3–79.5)	73.1 (68.3–78.2)	0.32
Median baseline PSA, µg/L (IQR)	28.6 (11.4–61.8)	31.2 (10.8–118.8)	26.8 (11.4–59.8)	0.49
ECOG performance status ≥ 1, n (%)	81 (36.7)	10 (37.0)	71 (36.6)	1
Median baseline ALP, U/L (IQR)	74.5 (58.8–127.8)	102.5 (57.5–193.5)	74 (59.0–118.8)	0.23
Bone metastases present, n (%)	181 (81.9)	22 (81.5)	159 (82.0)	1
Visceral metastases present, n (%)	10 (4.5)	2 (7.4)	8 (4.1)	0.36
Radical treatments prior to mCRPC, n (%)				0.62
Radical prostatectomy	16 (7.2)	2 (7.4)	14 (7.2)	
Radiation	6 (2.7)	1 (3.7)	5 (2.6)	
Radiation and endocrine	23 (10.4)	1 (3.7)	22 (11.3)	
None	175 (79.2)	23 (85.2)	152 (78.4)	
Other	1 (0.5)	0	1 (0.5)	
Taxane treatment prior to mCRPC, n (%)	65 (29.4)	8 (29.6)	57 (29.4)	1
First systemic therapy for mCRPC, n (%)				1
Enzalutamide	195 (88.2)	24 (88.9)	171 (88.1)	
Abiraterone	23 (10.4)	3 (11.1)	20 (10.3)	
Docetaxel	3 (1.4)	0	3 (1.5)	
Median time, ADT to mCRPC, months (IQR)	19.3 (11.9–38.3)	12.7 (7.0–22.5)	20.0 (12.7–39.0)	**0.03**
Median time, 1st Line ARSI, months (IQR)	10.6 (6.0–18.0)	8.4 (6.5–12.0)	10.8 (6.0–18.7)	0.46
Dead, n (%)	97 (43.9)	14 (51.9)	83 (42.8)	0.41

Significant values are in bold.

Twenty-seven patients (n = 27/221, 12.2%) carried a pathogenic or likely-pathogenic germline variant in a DNA repair gene (DRG carriers, Fig. 1B). Of these 27 DRG carriers, six had an *ATM* variant (2.7%), six had a *CHEK2* variant (2.7%), and three had a *BRCA2* variant (1.4%, Fig. 1B, Supplementary Table S2). No *BRCA1* variants were detected in this cohort.

### Clinical characteristics of DNA repair gene carriers and noncarriers

Clinical characteristics for the full patient cohort are summarized in Table 1. DRG carriers had significantly shorter median time from initiation of ADT to castration resistance compared to noncarriers (12.7 vs 20.0 months; *P* = 0.03, Table 1, Fig. 1a). Other patient characteristics at mCRPC baseline and at primary PC diagnosis were similar between DRG carriers and noncarriers (Table 1, Supplementary Table S3). Similar results were obtained when comparing baseline characteristics for patients carrying *BRCA2*/*ATM* variants specifically and noncarriers (Supplementary Table S4).

### Progression-free survival on first-line androgen signaling inhibitor treatment

**Figure 2:**
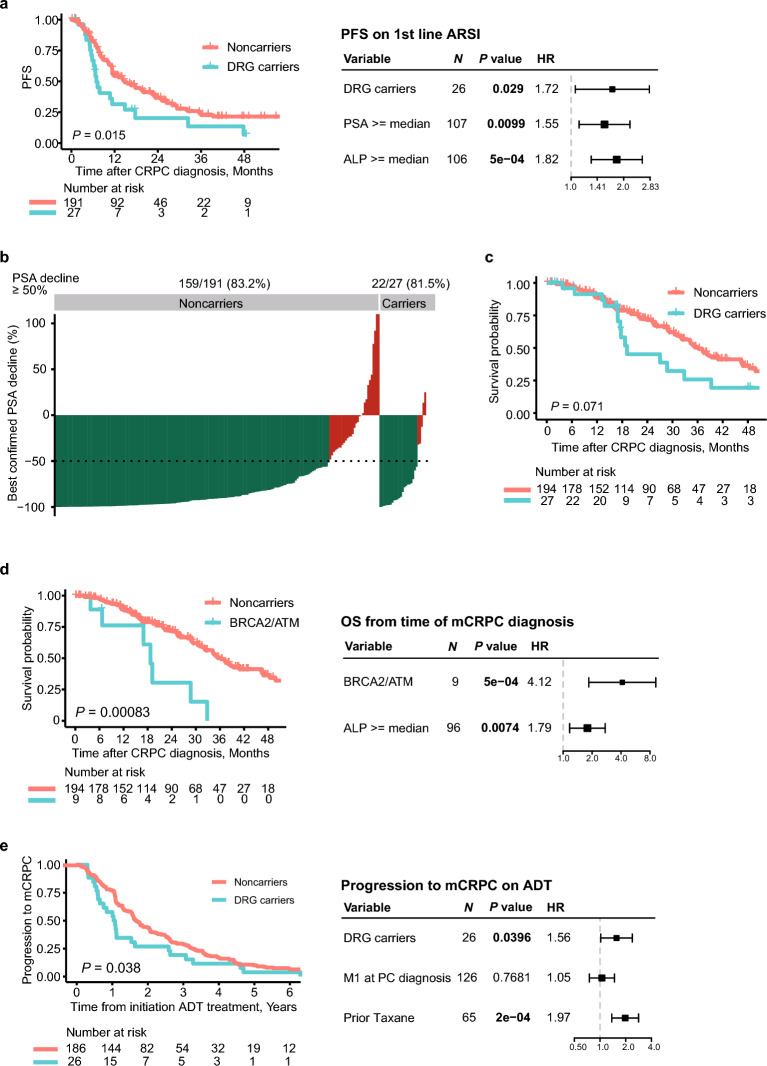
Outcomes of DRG carriers. Kaplan–Meier curves (left panel) and forest plots of multivariate cox regression analysis (right panel) for: (**a**) PFS on first-line ARSI; (**c**, **d**) OS from time of mCRPC diagnosis; and (**e**) progression to mCRPC on ADT. (**b**) Waterfall plot of best confirmed PSA change (PSA nadir) during first-line ARSI treatment in each patient. *DRG* DNA-repair genes, *ADT* Androgen deprivation therapy, *ALP* alkaline phosphate, *M1* metastases, *mCRPC* metastatic castration-resistant prostate cancer, *ARSI* androgen signaling inhibitor, *PFS* progression-free survival, *OS* overall survival, *PSA* prostate-specific antigen.

DRG carriers had significantly shorter progression-free survival (PFS) time on first-line ARSI compared with noncarriers (HR 1.80, 95% CI 1.12–2.89, *P* = 0.02, Fig. 2a). In multivariate cox regression analysis, DRG carrier status remained an independent prognostic predictor of PFS on first-line ARSI in mCRPC (HR 1.72, 95% CI 1.06–2.81, *P* = 0.029, Fig. 2a), after adjusting for significant baseline clinicopathological characteristics (Supplementary Fig. S1). Similar results were obtained when evaluating patients treated with enzalutamide separately (Supplementary Fig. S2).

### PSA response to first-line androgen signaling inhibitor treatment

We observed no significant differences in PSA response rates to first-line ARSI treatment between DRG carriers and noncarriers. PSA reductions of 50% were observed in 81.5% (n = 22/27) of DRG carriers and 83.2% (n = 159/191) of noncarriers (*P* = 0.79, Fig. 2b), while PSA reductions of 90% were observed in 44.4% (n = 12/27) and 51.8% (n = 99/191) (*P* = 0.54, data not shown), respectively. Similar results were obtained for the patient subgroup treated with enzalutamide (Supplementary Fig. S2). Thus, the poor PFS observed for DRG carriers on first-line ARSI seems not to be caused by worse treatment response compared to noncarriers, but by faster progression.

### Overall survival from time of mCRPC diagnosis

Overall survival (OS) was shorter among DRG carriers than noncarriers (median 17.6 vs 21.9 months, HR 1.68, 95% CI 0.95–2.96, *P* = 0.07, Fig. 2c), although this trend was not statistically significant in our moderately-sized cohort. For *BRCA2*/*ATM* specifically, carriers had significantly shorter OS compared with noncarriers (HR 3.51, 95% CI 1.60–7.71, *P* = 0.0017, Fig. 2d). Further, multivariate cox regression analysis identified *BRCA2*/*ATM* carrier status as an independent adverse prognostic predictor of OS from the time of mCRPC diagnosis (HR 4.12, 95% CI 1.85–9.19, *P* = 0.0005, Fig. 2d), after adjusting for significant baseline clinicopathological characteristics (Supplementary Fig. S1).

### Outcomes from time of initiation of androgen deprivation therapy

As retrospective information on clinicopathological characteristics and prior treatment received in the hormone-sensitive stage was available for all mCRPC patients, we further investigated the impact of germline DRG variants on patient outcomes at the time of starting primary ADT. DRG carriers had significantly shorter time to progression to mCRPC from initiation of ADT compared with noncarriers (HR 1.54, 95% CI 1.02–2.33,* P* = 0.04, Fig. 2e). Multivariate cox regression analysis identified DRG carrier status as an independent prognostic predictor for progression to mCRPC on ADT (HR 1.56, 95% CI 1.02–2.39, *P* = 0.04, Fig. 2e), after adjusting for significant clinicopathological characteristics (Supplementary Fig. S1).

In addition, OS from time of initiating ADT was significantly shorter among DRG carriers than noncarriers (HR 2.00, 95% CI 1.11–3.47, *P* = 0.02, Supplementary Fig. S3). Furthermore, in multivariate cox regression analysis, DRG carrier status was an independent adverse prognostic predictor for OS from time of initiating ADT (HR 1.99, 95% CI 1.21–3.52, *P* = 0.02, Supplementary Fig. S3), after adjusting for clinicopathological characteristics at primary diagnosis and upfront taxane treatment (Supplementary Fig. S1).

### Impact of individual DNA repair genes on patient outcomes

To better understand the relative contribution of specific germline DRG variants on mCRPC patient outcomes, we investigated the performance of individual DNA repair genes for predicting PFS on first-line ARSI, OS from time of mCRPC diagnosis, progression to mCRPC on ADT, and OS from time of initiating ADT, respectively. Genes with germline variants detected in at least three patients were evaluated individually (*CHEK2*, *ATM*, and *BRCA2*). Genes with germline variants in one or two patients were combined into one set of ‘Rare’ variants (Fig. 1b).

**Figure 3:**
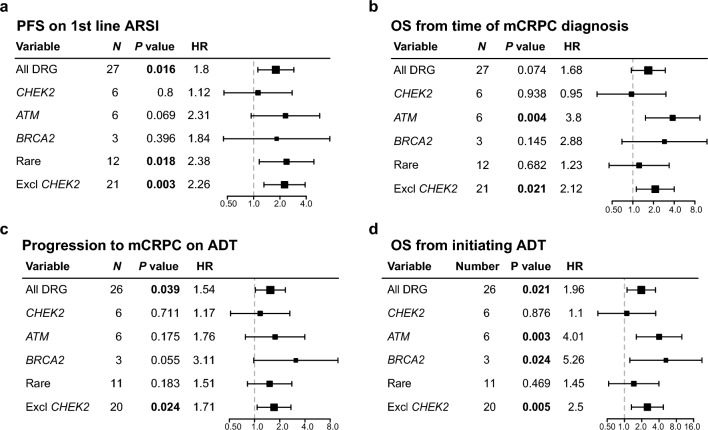
Impact of individual DNA repair genes on patient outcomes. Forest plots of univariate cox regression analysis for: (**a**) PFS on first-line ARSI; (**b**) OS from time of mCRPC diagnosis; (**c**) progression to mCRPC on ADT; and (**d**) OS from initiating ADT. *DRG* DNA-repair genes, *ADT* Androgen deprivation therapy, *mCRPC* metastatic castration-resistant prostate cancer, *ARSI* androgen signaling inhibitor, *PFS* progression-free survival, *OS* overall survival.

*CHEK2* variants were not associated with differences in outcome for any of the four clinical endpoints, showing HRs around 1 (0.95–1.17, Fig. 3). Thus, *CHEK2* variants seem not to contribute to the poor prognosis of DRG carriers identified in this study. *ATM* and *BRCA2* variants showed the strongest association to poor patient outcomes (Fig. 3). Interestingly, the ‘Rare’ variant group showed a relatively strong association to both shorter PFS and progression to mCRPC (Fig. 3).

**Figure 4:**
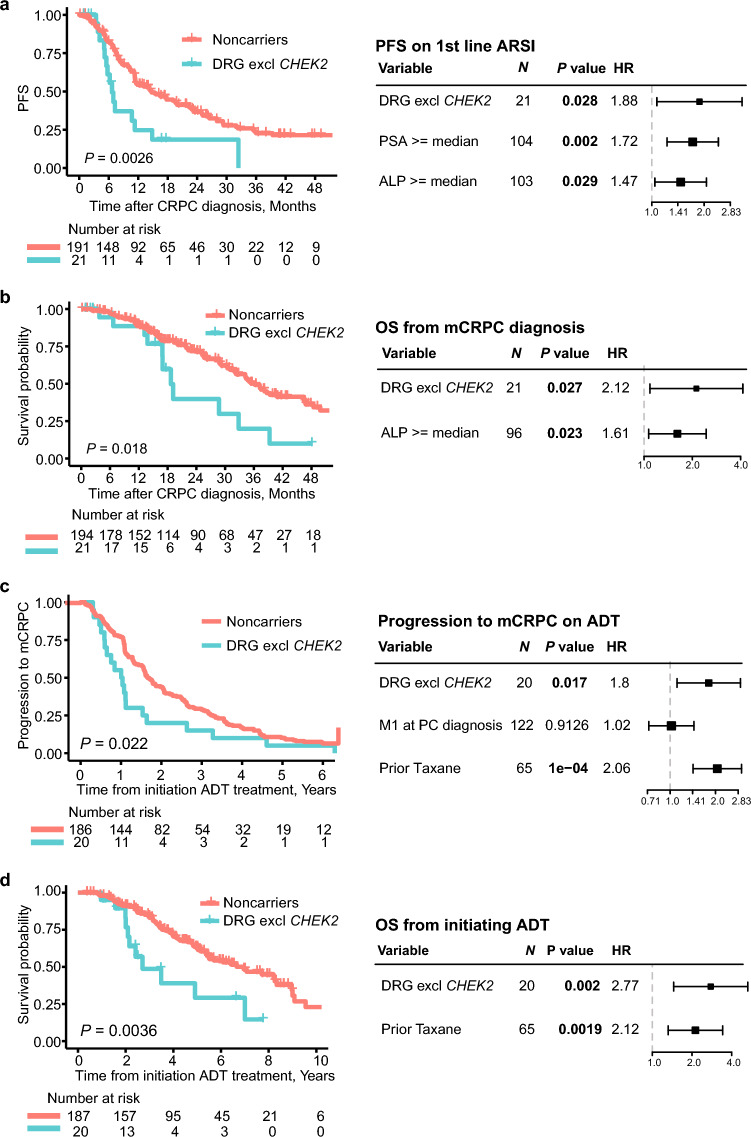
Outcomes of DRG carriers excluding *CHEK2* variants. Kaplan–Meier curves (left panel) and forest plots of multivariate cox regression analysis (right panel) for: (**a**) PFS on first-line ARSI; (**b**) OS from mCRPC diagnosis; (**c**) progression to mCRPC on ADT; and (**d**) OS from initiating ADT. *DRG*  DNA-repair genes, *ADT* Androgen deprivation therapy, *ALP* alkaline phosphate, *M1* metastases, *mCRPC* metastatic castration-resistant prostate cancer, *ARSI* androgen signaling inhibitor, *PFS* progression-free survival, *OS* overall survival.

Excluding *CHEK2* variants from the DRG carrier set, DRG carrier status was independently associated with PFS on first-line ARSI treatment, OS from mCRPC diagnosis, progression to mCRPC on ADT, and OS from initiating ADT and increased the HRs of DRG carrier status in all four analyses (Fig. 4).

### Treatment sequence

**Figure 5:**
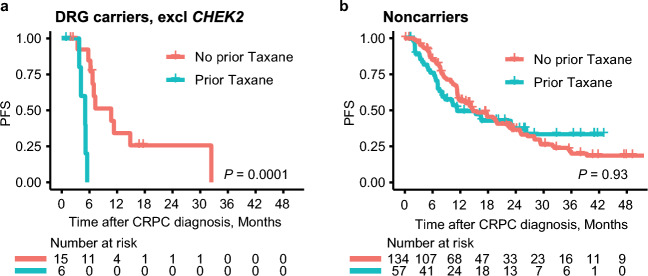
Outcomes of DRG carriers and noncarriers based on prior taxane treatment. (**a**, **b**) Kaplan–Meier curves for PFS on first-line ARSI treatment. *DRG* DNA repair genes, *PFS* progression-free survival on first-line androgen signaling inhibitor treatment.

A prior study suggested that outcomes of mCRPC germline *BRCA2* carriers may be modified by the initial treatment type, with the sequence of taxane-ARSI treatment negatively affecting *BRCA2* carriers, but not noncarriers^8^. Sixty-three patients in our cohort received upfront taxane treatment prior to mCRPC diagnosis. PFS on first-line ARSI treatment for DRG carriers, excluding *CHEK2* variants, was significantly shorter for carriers receiving prior taxane compared with carriers not receiving prior taxane treatment (5.1 vs 10.7 months, *P* = 0.0001, Fig. 5). This difference was not observed for noncarriers and could not be explained by the distribution of *BRCA2* and *ATM* variants (Supplementary Table S5).

## Discussion

In this study, we demonstrate an independent effect of germline DNA repair gene variants on the outcomes of patients with advanced PC. Despite carrier numbers being limited, we were able to identify germline DRG carrier status as an independent prognostic factor for PFS on first-line ARSI treatment, time to progression to mCRPC on ADT, and OS from initiating ADT. Furthermore, excluding *CHEK2* variants, germline DRG carrier status was an independent prognostic factor for OS from mCRPC diagnosis.

We screened 221 unselected mCRPC patients for germline DRG variants and identified 12.2% (n = 27/221) as carriers of a pathogenic variant, in line with previously reported carrier frequencies for this patient group (11.8–16.2%^5,8,9^). The prevalence of germline variants varies among populations and ethnic groups due to founder mutation effects^17,18^. *CHEK2* and *ATM* were the most frequently mutated genes in our cohort (both at 2.7%), both with slightly higher prevalence than previously reported (0.3–1.9% and 0.3–2%, respectively)^8,9,16^. *CHEK2* variants showed no significant association with patient outcomes in this cohort (Fig. 3). The founder mutation c.1100delC represented 67% (n = 4/6, Supplementary Table S2) of all *CHEK2* variants identified. In agreement with our results, this variant has previously been associated with PC predisposition, but not aggressiveness^11,19^.

Carriers of *ATM* variants were associated with worse outcomes for all clinical endpoints, with a significant association to poor OS both at time of initiation of ADT (HR 4.01, 95% CI 1.61–9.98, *P* = 0.003, Fig. 3d) and at time of mCRPC diagnosis (HR 3.80, 95% CI 1.53–9.46, *P* = 0.004, Fig. 3b). Germline *ATM* variants have previously been associated with aggressive and lethal PC^5,10,20^, however, to our knowledge, germline *ATM* variants have not previously been reported independent of *BRCA1/2* variants in survival analysis of mCRPC patient outcomes.

The prevalence of *BRCA2* variants (n = 3/221, 1.4%) was slightly lower in our cohort than previously reported for metastatic PC (2.9–5.3%^5,8,9,16,21^). Whether this attributes to a lower prevalence of germline *BRCA2* variants in the Danish population or reflects differences in variant interpretation protocols, i.e. adhering or not adhering to the ACMG classification guidelines, is unknown. Nevertheless, consistent with a previous study^8^, *BRCA2* carriers had shorter time to mCRPC progression on ADT (13.3 vs 19.9 months, *P* = 0.055, Fig. 3c; Castro et al.^8^: 13.2 vs 28.4 months, *P* = 0.048) and OS from mCRPC diagnosis (18.9 vs 36.2 months, *P* = 0.15, Fig. 3b; Castro et al.^8^: 17.4 vs 33.2 months; *P* = 0.027) compared to noncarriers. Further, *BRCA2*/*ATM* carrier status was an independent predictor of OS from mCRPC diagnosis (HR 4.12, 95% C: 1.85–9.19, *P* = 0.0005, Fig. 2d), in agreement with results from Na et al.^10^, who reported *BRCA1*/*2* and *ATM* carrier status to be a significant predictor of poor cause-specific survival in a cohort of localized and metastatic PC patients.

Of the genes with germline variants detected in fewer than three patients, i.e. the ‘Rare’ variants (Fig. 1), *NBN* has previously been associated with aggressive disease and poor survival in PC patients^22,23^. Less is known about the role in PC of germline variants in the remaining genes of the ‘Rare’ variant group (*MUTYH*, *RAD50*, *NTHL1*, *RECQL4*, *FANCM*, *ERCC6*, *FANCA*, and *PNKP)*. Interestingly, in our study, the combined set of these ‘Rare’ variants showed a relatively strong association to poor patient outcome, especially for PFS on first-line ARSI and progression to mCRPC on ADT (HR 2.38, *P* = 0.018; HR 1.51, *P* = 0.18, respectively, Fig. 3), supporting the impact of multiple DNA repair genes beside *BRCA2* and *ATM* on the poor prognosis of this patient group. Further studies are needed to fully establish the influence of these rare and less-known germline DRG variants on the prognosis and treatment of mCRPC patients.

To date, studies describing the impact of germline DRG variants on first-line ARSI treatment of mCRPC patients have been conflicting. Here, we identified DRG carriers to have shorter PFS on first-line ARSI treatment compared with noncarriers, independent of baseline mCRPC clinical parameters (Fig. 2a). This is in line with the results of Annala et al.^16^, who reported the outcomes of 176 mCRPC patients treated with an ARSI, including 21 germline DRG carriers, and who identified DRG carriers to have significantly shorter PFS on first ARSI compared with noncarriers (3.3 vs 6.2 months, *P* = 0.01).

The PROREPAIR-B study reported a trend towards shorter PFS for mCRPC patients on first ARSI (not limited to first-line treatment) for germline DRG carriers compared with noncarriers (n = 365), although this was not statistically significant (8.1 vs 9.2 months, *P* = 0.59)^8^. A possible reason the PROREPAIR-B study did not identify a significant association could be the PFS analysis included patients receiving first ARSI treatment, but not necessarily first-line of mCRPC treatment. However, additional validation is still warranted.

In addition, the PROREPAIR-B study found the outcomes of germline *BRCA2* carriers to be modified by the sequence of ARSI and taxane treatment given in their mCRPC cohort. They identified *BRCA2* carriers receiving the sequence of taxane-ARSI to have significantly worse cause-specific survival and PFS than noncarriers receiving the same treatment. In contrast, no difference was observed when investigating carriers and noncarriers receiving ARSI-taxane sequence.

In our cohort, only three patients received a taxane as first-line mCRPC treatment. However, 63 patients received a taxane prior to mCRPC diagnosis, in the setting of initial ADT. We observed DRG carriers receiving upfront taxane treatment prior to first-line ARSI mCRPC treatment to have significantly shorter PFS on first-line ARSI than DRG carriers not receiving upfront taxane treatment (5.1 vs 10.7 months, *P* = 0.0001, Fig. 5), whereas no difference in PFS was observed for noncarriers. Accordingly, although taxane in our study was given upfront and not in the mCRPC setting, our results support the hypothesis that the sequence of taxane-ARSI negatively affects DRG carriers, but not noncarriers. However, additional prospective data are needed to shed further light on this issue.

Our findings suggest DRG carriers to have a worse prognosis than noncarriers. Consequently, DRG carriers may benefit from intensified treatment, likely already at the hormone-sensitive stage of PC. Accordingly, positive DRG carrier status could serve as an additional risk factor beyond current clinicopathological parameters to guide the treatment of hormone-sensitive PC patients. This may potentially include selection for emerging PARP inhibitors, which are being investigated in ongoing randomized control trials in combination with standard of care in patients with hormone-sensitive PC (ClinicalTrials.gov ID: NCT04947254, NCT04821622, NCT05498272). However, additional studies are needed to investigate if DRG carriers would benefit from such intensified treatment. Our findings further indicate that additional DNA repair genes besides *BRCA1/2* and *ATM* may be included in germline testing of metastatic PC patients, as other DNA repair genes showed a strong association to worse patient outcomes (Figs. 3, 4). Nonetheless, additional studies are needed before a change in clinical practice may be recommended. Lastly, our results suggest that the treatment sequence might impact the outcomes of germline DRG carriers and that it may be preferable to avoid the treatment sequence of taxane-ARSI for germline DRG carriers, in agreement with prior finding^8^. However, larger studies specifically designed to address this objective are needed.

We acknowledge the limitations of this study. First, the evaluation of patient outcomes, and particularly the prognostic impact of specific DNA repair genes on patient outcomes, is limited by small carrier numbers (n = 27). Therefore, the impact of DRG variants, including the missing impact of *CHEK2* variants, on mCRPC patient outcomes should be validated in larger cohorts. However, the missing impact of *CHEK2* variants on patient outcomes was consistently observed for all four clinical endpoints in this study and also supported by prior findings in other studies. Secondly, we did not analyze concurrent somatic DRG variants potentially present in a substantial proportion of noncarriers, and further potentially affecting the grade of DNA repair gene impairment in the DRG carrier group due to somatic loss of the second allele.

In conclusion, in this study, we have demonstrated an independent effect of germline DRG variants on PFS and OS for mCRPC patients, including outcomes from initiation of primary ADT at the hormone-sensitive stage of PC. Our findings suggest that multiple other DNA repair genes besides *ATM* and *BRCA2* influence the outcomes of advanced PC DRG carriers. Additional validation is required before a change in clinical practice is supported.

## Patients and methods

### Patients

This study included 221 men with mCRPC, who were diagnosed and received first-line mCRPC treatment between January 2016 to October 2021 at Aarhus University Hospital or The Regional Hospital of West Jutland, Denmark. Patients were recruited consecutively and were not selected based on prior knowledge of germline/somatic mutations, age at diagnosis, and/or familial cancer history. All research was carried out in accordance with relevant guidelines and regulations. The study was approved by The National Committee on Health Research Ethics (#1901101) and notified to the Danish Data Protection Agency (#1-16-02-366-15). All patients provided written informed consent for biobanking. The requirement for patient consent to the specific analyses in this study was waived.

### Study design

All patients were prospectively enrolled at the time of mCRPC diagnosis immediately before starting first-line mCRPC treatment. Clinicopathological characteristics and outcomes of mCRPC disease were collected prospectively, whereas clinical information on primary PC diagnosis and prior treatments of localized and/or advanced disease in the hormone-sensitive stage was collected retrospectively. Patients generally had PSA measurements every 3 months and were followed until death or withdrawal from the study.

### Sample collection and processing

Germline DNA was extracted from whole blood collected in 10 mL BD Vacutainer K_2_ EDTA tubes (Beckton Dickinson) and kept cool (4 °C) until processing. EDTA-blood samples were centrifuged at 3000×*g* for 10 min at 20 °C to extract the cellular components (buffy coat). Buffy coat was transferred to a 1.5 mL cryotube and stored at − 80 °C until DNA extraction. Centrifugation, processing, and storage was done within 3 h.

Germline DNA was extracted on a QIAsymphony robot (Qiagen) using the QiaSymphony DSP DNA Mini Kit (Qiagen) according to manufacturer’s instructions and previously described^24^. DNA concentrations were determined using Qubit fluorometric quantification (dsDNA Broad range, ThermoFisher). Eluates were stored at − 20 °C until further analysis.

### Target capture sequencing

Germline DRG variants were detected using targeted exon capture sequencing of a custom panel including 114 DNA repair genes (Suppl. Table S1). A SureSelect XT custom bait library was designed for coding regions (plus 50 base pair (bp) exon flanking regions) of each of the 114 DNA repair genes (SureDesign, Agilent Technologies). Libraries were prepared using NEBNext Ultra II FS DNA library kit and in-house target enrichment^11^. In brief, for each specimen, 600 ng of DNA underwent enzymatic fragmentation (200–350 bp), adaptor ligation, size selection of adaptor-ligated DNA, and a polymerase chain reaction (PCR) amplification (6 cycles) of adaptor-ligated DNA (NEBNext Ultra FS II DNA Library Prep kit for Illumina, cat #E7805L and NEBNext Multiplex Oligos for Illumina (96 Index Primers) #E6609S). After a clean-up of PCR amplification, library QC was assessed on a Bioanalyzer (DNA1000 Bioanalyzer kit, cat. #5067-1504, Agilent Technologies) and quantified by PicoGreen using a Quant-iT PicoGreen dsDNA Assay Kit (Invitrogen, cat # P7589). Samples were hereafter pooled in batches of 16 with a combined quantity of 1 μg and hybridized to a single SureSelect XT Custom bait library for 48 h at 65 °C. The hybridized libraries were hereafter captured, washed, and amplified by PCR using NEBNext Ultra II Q5 Master Mix (#M0544). Final libraries were purified with Dynal MyOne Streptavidin T1 beads and assessed by Bioanalyzer (High Sensitivity Bioanalyzer kit, cat. #5067-4626, Agilent Technologies) and quantified by qPCR using NEBNext Library Quant Kit for Illumina (cat #E7630L). Samples were pooled and ensured a concentration of 0.5–2 nM by Qubit (dsDNA HS assay kit, #Q32851, Invitrogen). Pooled captured libraries were sequenced using Illumina MiSeq kit 150 cycle v3 (#MS-102-3001m, Illumina). Median read depth in analyzable target regions across all patients was 58.

### Germline variant annotation

Sequencing files were processed with the Genome Analysis Toolkit pipeline (GATK v4.0)^25^ following GATK best practice recommendations for germline single nucleotide variant and indel calling^26,27^, as well as Agilent SureCall (v4.2). Variants from GATK were annotated using the Variant Effect Predictor (VEP v101^28^).

Samples were retained if ≥ 80% of the captured regions were sequenced at a minimum 20 × read depth and if ≥ 80% reads were mapped to captured regions. Samples failing established quality control (n = 6/221) were resequenced, after which all samples passed QC. We removed low-quality variants with a read-depth < 20, those in repetitive regions (simple repeats, segmental duplications, and centromeric regions), heterozygous variants with an allelic ratio < 30% or > 70%, and those for which the 1000 Genomes Project minor allele frequency in any population was > 1%^29^.

Variants analyzed either had a predicted impact of “HIGH” or “MODERATE” or a pathogenic/likely pathogenic ClinVar classification. The pathogenicity of detected germline variants was determined according to the American College of Medical Genetics and Genomics guidelines^30^ with the adaptions of the Cancer Variant Interpretation Group UK^31^. At least two independent reviewers evaluated all variants against published literature, variant databases, including ClinVar, in silico predictions, and population frequency databases. The Non-Finnish European genome aggregation database (gnomAD) cohort was used for variant minor allele frequency comparisons^32^. Variants classified as pathogenic or likely-pathogenic were included in the study.

### Clinical outcomes

The primary endpoint was PFS on first-line ARSI treatment, which was defined as the time (in months) from therapy initiation until PSA progression or death from any cause, whichever was observed first. In cases with initial PSA decrease after treatment initiation, PSA progression was defined by an absolute increase in PSA ≥ 2 ng/mL and reaching a PSA level > 25% higher than PSA nadir. In cases with no initial PSA decrease, PSA progression was defined as an absolute increase ≥ 2 ng/mL as compared to baseline PSA and > 25% increase in total PSA level at least 12 weeks after treatment initiation. PSA progression was confirmed by a second sample ≥ 3 weeks later, as recommended by the Prostate Cancer Clinical Trials Working Group [PCWG3]^33^.

For PSF analysis, patients who did not experience PSA progression or death were censored at the time of their last PSA measurement or at the end of treatment.

ARSI treatment response was evaluated by PSA response rates, defined as the best confirmed PSA response during treatment (PSA nadir). Secondary endpoints included (i) progression to mCRPC on ADT, defined as the time from initiation of continuous ADT to progression to mCRPC, (ii) OS from mCRPC diagnosis, defined as the time from mCRPC diagnosis to death from any cause, and (iii) OS from initiation of ADT, defined as the time from initiation of continuous ADT to death from any cause. For OS analysis, those patients who remained alive at the time of database lock were censored at the last follow-up. The last date of follow-up (database lock) was December 5th, 2022.

### Statistical analysis

Baseline clinicopathological characteristics at primary PC and at mCRPC diagnosis for carriers of a pathogenic or likely-pathogenic DRG variant (DRG carriers) were compared against those of patients without a pathogenic or likely-pathogenic DRG variant (noncarriers) using Wilcoxon rank sum test for continuous variables or Fisher’s exact test for categorical variables.

Time-to-event endpoints were evaluated using Kaplan–Meier analysis, the log-rank test, and uni- and multivariate cox regression analyses using the survival package in R. The prognostic effect of DRG carrier status for predicting PFS, OS, and progression to mCRPC was adjusted for significant clinicopathological characteristics in multivariate cox regression. Clinicopathological characteristics adjusted for included metastasis status at primary PC diagnosis, prior taxane therapy, alkaline phosphatase (ALP) ≥ median cohort value (74 U/L), and PSA ≥ median cohort value (28.6 µg/L). For all cox regression analyses, the proportional hazards assumption was tested. For all endpoints, germline DRG carrier status fulfilled the proportionality criteria. All clinical variables, except for prior taxane treatment and metastasis status in analyses of progression to mCRPC, fulfilled the proportionality criteria.

All statistical tests were two-sided. Statistical analyses were performed using R v.3.4.0^34^.

### Supplementary Information

Supplementary Information.

## Data Availability

The data generated and analyzed for this study is available through controlled access from GenomeDK (https://genome.au.dk/) under accession number GDK000006 (https://genome.au.dk/library/GDK000006/).
