# Supplementary material for: Impact of germline DNA repair gene variants on prognosis and treatment of men with advanced prostate cancer
Source: Sci Rep. 2023 Nov 6;13:19135. doi: 10.1038/s41598-023-46323-5 (PMC10628129; doi:10.1038/s41598-023-46323-5)
Supplement: Supplementary file 1 — Supplementary Information. [file 41598_2023_46323_MOESM1_ESM.pdf]

Supplementary Information

Impact of Germline DNA Repair Gene Variants on Prognosis and Treatment of Men with Advanced Prostate Cancer

Emma B. Hansen, Questa Karlsson, Susan Merson, Sarah Wakerell, Reshma Rageevakumar, Jørgen B. Jensen, Michael Borre, Zsofia Kote-Jarai[†], Rosalind A. Eeles[†], Karina D. Sørensen[†].

[†]These authors are joint last authors.

Supplemental Figures

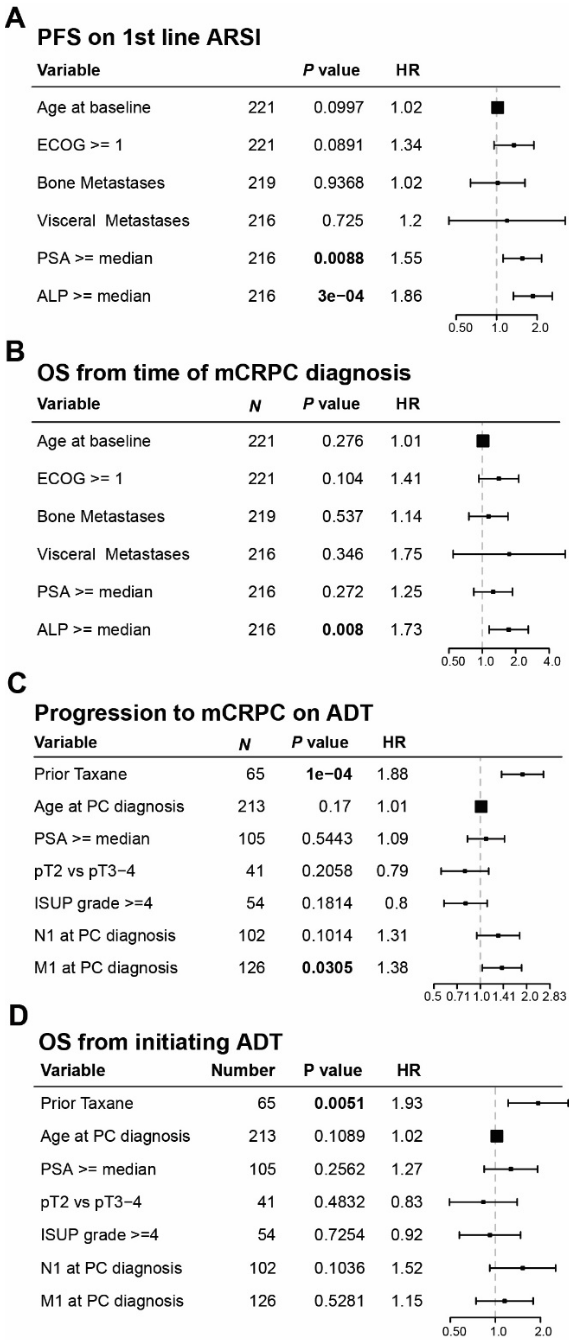

Suppl. Fig. S1. Performance of clinicopathological characteristics on patient outcomes. Forest plots of univariate cox regression analysis for: **(A)** PSF on first-line ARSI; (**B**) OS from time of mCRPC diagnosis; (**C**) progression to mCRPC on ADT; and (**D**) OS from initiating ADT. DRG = DNA-repair genes, ADT = Androgen deprivation therapy, ALP = alkaline phosphate, ECOG = Eastern Cooperative Oncology Group, N1 = lymph nodes, M1 = metastases, mCRPC = metastatic castration-resistant prostate cancer, ARSI = androgen signaling inhibitor, PSF = progression-free survival, OS = overall survival.

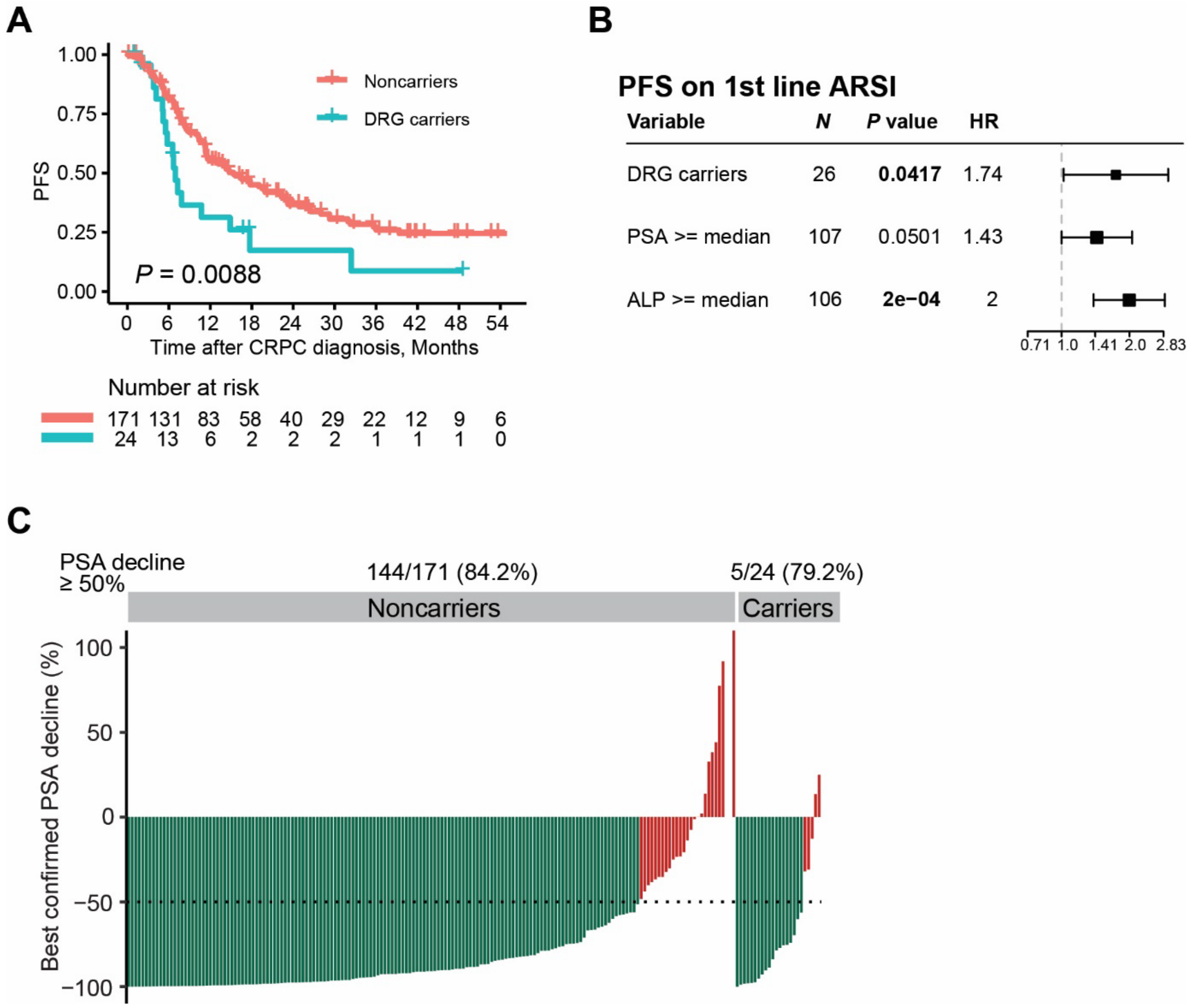

Suppl. Fig. S2. Outcomes of DRG carriers for patients treated with Enzalutamide. A-B) Kaplan-Meier curve and forest plot of multivariate cox regression analysis for PSF on first-line enzalutamide treatment. **C)** Waterfall plot of best confirmed PSA change during first-line enzalutamide treatment. DRG = DNA-repair genes, ALP = alkaline phosphate, PSF = progression-free survival, PSA = prostate-specific antigen.

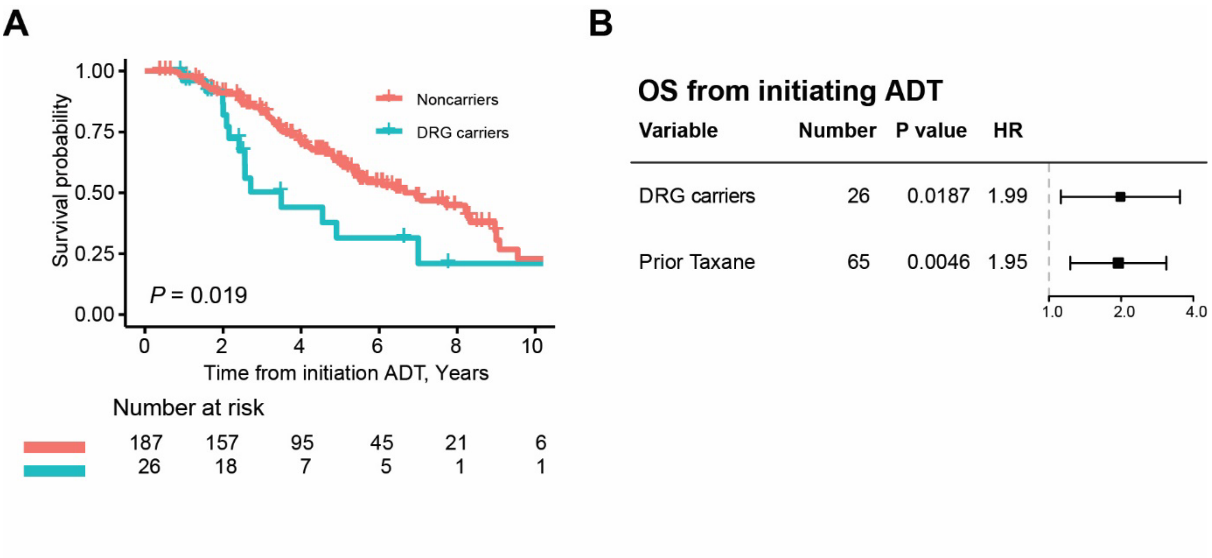

Suppl. Fig. S3. OS from time of initiating ADT. A-B) Kaplan-Meier curve and forest plot of multivariate cox regression analysis for OS from initiation of primary ADT. DRG = DNA-repair genes, ADT = androgen deprivation therapy, OS = overall survival.

Supplemental Tables

Suppl. Table S1. DNA-Repair Genes

ALKBH3	*DCLRE1A*	*MLH1*	*POLD1*	*SETMAR*
APEX1	*EME1*	*MLH3*	*POLE*	*SLX4*
AR	*EME2*	*MMS19*	*POLK*	*SMAD4*
ATM	*ERCC2*	*MNAT1*	*POLM*	*SMARCA4*
ATR	*ERCC5*	*MPG*	*POLN*	*SMUG1*
ATRIP	*ERCC6*	*MRE11A*	*POLQ*	*SPOP*
BAP1	*ESR2*	*MSH2*	*POT1*	*STK11*
BARD1	*EXO1*	*MSH5*	*PRSS1*	*TDG*
BLM	*FAM175A*	*MSH6*	*PTCH1*	*TOP2A*
BRCA1	*FANCA*	*MSR1*	*PTEN*	*TOP2B*
BRCA2	*FANCD2*	*MUTYH*	*RAD1*	*TOP3A*
BRIP1	*FANCI*	*NABP2*	*RAD50*	*TP53*
CCNH	*FANCL*	*NBN*	*RAD51B*	*TP53BP1*
CDC25C	*FANCM*	*NEIL1*	*RAD51C*	*WRN*
CDH1	*GADD45A*	*NEIL2*	*RAD51D*	*XAB2*
CDK4	*GEN1*	*NTHL1*	*RAD52*	*XPA*
CDKN2A	*GTF2H2*	*OGG1*	*RAD54B*	*XPC*
CHD1	*GTF2H3*	*PALB2*	*RAD54L*	*XRCC1*
CHEK1	*GTF2H4*	*PARP2*	*RB1*	*XRCC2*
CHEK2	*HUS1*	*PER1*	*RECQL*	*XRCC4*
CLK2	*LIG1*	*PMS1*	*RECQL4*	*XRCC5*
	LIG3	*PMS2*	*RECQL5*	
	LIG4	*PNKP*	*RINT1*	
			RNASEL	
			RPA1	

Suppl. Table S2. Pathogenic/likely-pathogenic germline variants detected in mCRPC cohort.

Gene	Chr	Pos	Ref	Alt	HGVS	Type	Effect	Class
ATM	11	108202280	ATAATG	A	c.7626_7629+1delTAATG	Deletion	FrameShift	LP
ATM	11	108202280	ATAATG	A	c.7626_7629+1delTAATG	Deletion	FrameShift	LP
ATM	11	108196200	TGTATTAAGGACATTCTCAC	CA	c.6736_6755delinsCA (p.Cys2246_Thr2252delinsHis)	Indel	Inframe deletion	LP
ATM	11	108173586	G	T	c.5326G>T (p.Glu1776*)	SNV	Nonsense	Pathogenic
ATM	11	108186742	C	T	c.6100C>T (p.Arg2034*)	SNV	Nonsense	Pathogenic
ATM	11	108224608	G	A	c.8786+1G>A	SNV	SpliceSite	Pathogenic
BRCA2	13	32914209	ACT	A	c.5722_5723delCT (p.Leu1908Argfs*2)	Deletion	FrameShift	Pathogenic
BRCA2	13	32937327	A	T	c.7988A>T (p.Glu2663Val)	SNV	Missense	Pathogenic
BRCA2	13	32937326	G	A	c.7987G>A (p.Glu2663Lys)	SNV	Missense	LP
CHEK2	22	29091856	AG	A	c.1100del (p.Thr367Metfs*15)	Deletion	FrameShift	Pathogenic
CHEK2	22	29091856	AG	A	c.1100del (p.Thr367Metfs*15)	Deletion	FrameShift	Pathogenic
CHEK2	22	29091856	AG	A	c.1100del (p.Thr367Metfs*15)	Deletion	FrameShift	Pathogenic
CHEK2	22	29091856	AG	A	c.1100del (p.Thr367Metfs*15)	Deletion	FrameShift	Pathogenic
CHEK2	22	29090054	G	A	c.1427C>T (p.Thr476Met)	SNV	Missense	LP
CHEK2	22	29090054	G	A	c.1427C>T (p.Thr476Met)	SNV	Missense	LP
ERCC6	10	50740782	G	A	c.229C>T (p.Arg77Ter)	SNV	Nonsense	Pathogenic
FANCA	16	89858441	GCCAA	G	c.1115_1118delTTGG (p.Val372Alafs*42)	Deletion	FrameShift	Pathogenic
FANCM	14	45618130	A	T	c.850A>T (p.Arg284*)	SNV	Nonsense	LP
MUTYH	1	45797228	C	T	c.1103G>A (p.Gly368Asp)	SNV	Missense	Pathogenic
NBN	8	90955480	C	A	c.2184+1G>T	SNV	SpliceSite	LP
NTHL1	16	2096239	G	A	c.244C>T (p.Gln82Ter)	SNV	Nonsense	Pathogenic
NTHL1	16	2096239	G	A	c.244C>T (p.Gln82Ter	SNV	Nonsense	Pathogenic
PNKP	19	50365032	GCGCGGCT	G	c.1288_1294delAGCCGCG (p.Ser430Profs*35)	Deletion	FrameShift	Pathogenic
RAD50	5	131939076	T	TA	c.2165dupA (p.Glu723Glyfs*5)	Insertion	FrameShift	Pathogenic
RAD50	5	131931451	AAAGAACGACATAG	A	c.2299_2311delGACATAGAAGAAC (p.Asp767Lysfs*8)	Deletion	FrameShift	LP
RECQL4	8	145741453	CCT	C	c.1048_1049delAG (p.Arg350Glyfs*21)	Deletion	FrameShift	Pathogenic
RECQL4	8	145738395	G	A	c.2590C>T (p.Gln864*)	SNV	Nonsense	Pathogenic

SNV = single nucleotide variant, Indel = Small insertions and deletions, LP = Likely Pathogenic

Suppl. Table S3. Patient characteristics at primary prostate cancer diagnosis

Clinical variable		Full cohort (*N*=221)	Carriers (*N*= 27)	Noncarriers (*N*= 194)	***P* value**
Median age at diagnosis (IQR)		69.1 (63.7-74.5)	72.4 (65.0-76.6)	68.9 (63.3-73.5)	0.09
Median PSA at diagnosis (IQR)		53.5 (18.8-196.3)	32.5 (15.1-179.1)	54 (20.1-222)	0.26
ISUP grade ≥4 at diagnosis, n (%)		153 (69.2)	20 (74.1)	133 (68.6)	1
T stage at diagnosis, n (%)					0.19
	T1/T2	42 (19.0)	8 (29.6)	34 (17.5)	
	T3/T4	166 (75.1)	18 (66.7)	148 (76.3)	
Localized disease at diagnosis, n (%)		34 (15.4)	2 (7.4)	32 (16.5)	0.38
N1 disease at diagnosis, n (%)		104 (47.1)	13 (48.1)	91 (46.9)	0.62
M1 disease at diagnosis, n (%)		128 (57.9)	17 (63.0)	111 (57.2)	0.52

Suppl. Table S4. Patient baseline characteristics and summary of prior and current treatments for localized, advanced, and castration-resistant prostate cancer for *BRCA2/ATM* carriers

Clinical variable	Full cohort (*N*=221)	BRCA2/ATM Carriers (*N*= 9)	Noncarriers (*N*= 194)	*P* value
Median age at diagnosis (IQR)	69.1 (63.7-74.5)	73.6 (67.4-75.6)	68.9 (63.3-73.5)	0.12
Median PSA at diagnosis (IQR)	53.5 (18.8-196.3)	66.9 (23.5-154.0)	54 (20.1-222)	1
ISUP group ≥4 at diagnosis, n (%)	153 (69.2)	7 (77.8)	133 (68.6)	1
T stage at diagnosis, n (%)				1
T1/T2	42 (19.0)	1 (11.1)	34 (17.5)	
T3/T4	166 (75.1)	7 (77.7)	148 (76.3)	
Localized disease at diagnosis, n (%)	34 (15.4)	1 (11.1)	32 (16.5)	1
N1 disease at diagnosis, n (%)	104 (47.1)	4 (44.4)	91 (46.9)	0.65
M1 disease at diagnosis, n (%)	128 (57.9)	4 (44.4)	111 (57.2)	0.72
Median age at baseline (IQR)	73.2 (68.5-78.5)	79 (70.3-79.5)	73.1 (68.3-78.2)	0.29
Median baseline PSA (IQR)	28.6 (11.4-61.8)	40.3 (5.6-90.5)	26.8 (11.4-59.8)	0.91
ECOG performance status ≥1, n (%)	81 (36.7)	4 (44.4)	71 (36.6)	0.73
Median baseline alkaline phosphatase (IQR)	74.5 (58.8-127.8)	68.0 (57.0-93.0)	74 (59.0-118.8)	0.39
Bone metastases present, n (%)	181 (81.9)	0	159 (82.0)	1
Visceral metastases present, n (%)	10 (4.5)	0	8 (4.1)	0.35
Radical treatments prior to mCRPC, n (%)				0.86
Radical Prostatectomy	16 (7.2)	1 (11.1)	14 (7.2)	
Radiation	6 (2.7)	0	5 (2.6)	
Radiation and Endocrine	23 (10.4)	1 (11.1)	22 (11.3)	
None	175 (79.2)	7 (77.8)	152 (78.4)	
Other	1 (0.5)	0	1 (0.5)	
Taxane treatment prior to mCRPC, n (%)	65 (29.4)	2 (22.2)	57 (29.4)	1
First systemic therapy for mCRPC, n (%)				0.35
Enzalutamide	195 (88.2)	7 (77.8)	171 (88.1)	
Abiraterone	23 (10.4)	2 (22.2)	20 (10.3)	
Docetaxel	3 (1.4)	0	3 (1.5)	
Median time ADT to mCRPC (IQR)	19.3 (11.9-38.3)	9.1 (7.2-13.4)	20.0 (12.7-39.0)	**0.04**
Median time 1st Line ARSI (IQR)	10.6 (6.0-18.0)	8.4 (7.0-12.6)	10.8 (6.0-18.7)	0.55
Dead, n (%)	97 (43.9)	7 (77.7)	83 (42.8)	0.08

Suppl. Table S5. Distribution of germline variants across patients receiving prior or no prior taxane.

Gene	Prior Taxane	No prior taxane
ATM	1	5
BRCA2	1	2
FANCM	0	1
FANCA	1	0
ERCC6	1	0
MUTYH	0	1
NBN	0	1
NTHL1	1	1
PNKP	0	1
RAD50	1	1
RECQL4	0	2
